# The complete chloroplast genome of *Cannabis sativa* variety Yunma 7

**DOI:** 10.1080/23802359.2021.1873709

**Published:** 2021-02-11

**Authors:** Gang Deng, Mei Yang, Kaiyuan Zhao, Yang Yang, Xing Huang, Xia Cheng

**Affiliations:** a School of Agriculture, Yunnan University, Kunming, China; b College of Agriculture and Life Sciences, Kunming University, Kunming, China; c Environment and Plant Protection Institute, Chinese Academy of Tropical Agricultural Sciences, Haikou, China

**Keywords:** *Cannabis sativa;* chloroplast genome, phylogenetic tree

## Abstract

*Cannabis sativa* variety Yunma 7 has been widely cultivated for hemp fiber production in Yunnan Province of China. In the present study, we have successfully sequenced the chloroplast genome of Yunma 7. The complete chloroplast genome size is 153,899 bp in length with a GC content of 36.67%. The genome contains a large single copy region (LSC) of 84,046 bp, a small single copy region (SSC) of 17,831 bp, and a pair of inverted repeat regions (IR) of 26,011 bp. A total of 74 protein-coding genes are annotated in the chloroplast genome, together with 37 tRNAs and 8 rRNAs. Phylogenetic tree reveals that Yunma 7 is closely related to *Cannabis* hybrid AK Royal Automatic reported by Matielo et al.

As the important raw materials for textile industry, hemp (*Cannabis sativa* L.) has been cultivated for thousands of years (Deng et al. 2019). It is also used for the production of nutritious seed, medicine, and recreational drug (Marks et al. 2009, Measham et al. 1994). The dried flowers and leaves of hemp are also used as narcotic materials, which caused its cultivation and products are illegal in many countries (Kojoma et al., 2006). In China, the cultivation of hemp cultivars with low (<0.3%) THC (tetrahydrocannabinol) concentration are allowed, which is also called industrial hemp. In recent years, *C. sativa* variety Yunma 7 has been widely cultivated as a main cultivar in Yunnan Province, one of the main production areas. However, little is known about the systematic position and phylogenetic relationship of Yunma 7 at cp genome level. In order to reveal its phylogenetic relationship in *Cannabis* genus, we selected next-generation sequencing to assemble the complete cp genome of Yunma 7.

The leaves of Yunma 7 were collected from the greenhouse of the School of Agriculture (24.83°N, 102.85°E), Yunnan University, Kunming, China. The total genomic DNA was extracted with the modified CTAB method (Doyle and Doyle 1987). The specimen was deposited in Herbarium of Yunnan University (HGS-dm2020004). Total DNA was sent to Biozeron Biotech (Shanghai, China) for library construction and next-generation sequencing. The Illumina NovaSeq platform was selected for sequencing with the generation of 0.91 Gb short reads data, which was then submitted to SRA under the accession number PRJNA679189. The trimmed reads were selected for cp genome assembly by NOVOPlasty software (Dierckxsens et al. 2017). The GapCloser software was used for gap filling (Luo et al. 2012). The assembled cp genome was annotated and corrected by DOGMA and Geneiousv11.0.3, respectively (Wyman et al. 2004; Kearse et al. 2012). The complete cp genome sequence was submitted to GenBank under the accession number MW013540.

The complete chloroplast genome size is 153,899 bp in length and the GC content is 36.67%. A large single copy region (LSC) of 84,046 bp, a small single copy region (SSC) of 17,831 bp, and a pair of inverted repeat regions (IR) of 26,011 bp are identified in the cp genome. A total of 74 protein-coding genes are annotated in the chloroplast genome, together with 37 tRNAs and 8 rRNAs. Among these, duplication events occurred with 12 genes (*ndhB*, *rps7*, *rps12*, *rps19*, *rpl2*, *rpl23*, *ycf1*, *ycf2*, *rrn4.5*, *rrn5*, *rrn16* and *rrn23*).

The cp genome sequences of 23 species were selected for phylogenetic analysis (19 species in Cannabaceae, 3 species in Moraceae and 1 specie in Ulmaceae as outgroup including *Morus mongolica*, *Morus indica*, *Morus notabilis* and *Ulmus macrocarpa*) (Oh et al. 2016; Vergara et al. 2016; Zhang et al. 2018; Matielo et al. 2020). The result indicated that Yunma 7 is closely related with *Cannabis* hybrid AK Royal Automatic reported by Matielo et al. (Figure 1). The two samples were both collected in Yunnan indicated that they might share similar genetic backgroud. MAFFT software was used for the alignment of cp genome sequences (Katoh and Standley 2013). A neighbor-joining phylogenetic tree was constructed in MEGA7 software with 100 bootstrap replicates (Kumar et al. 2016). This study will benefit future studies related to chloroplast in *Cannabis* genus.

**Figure 1.**
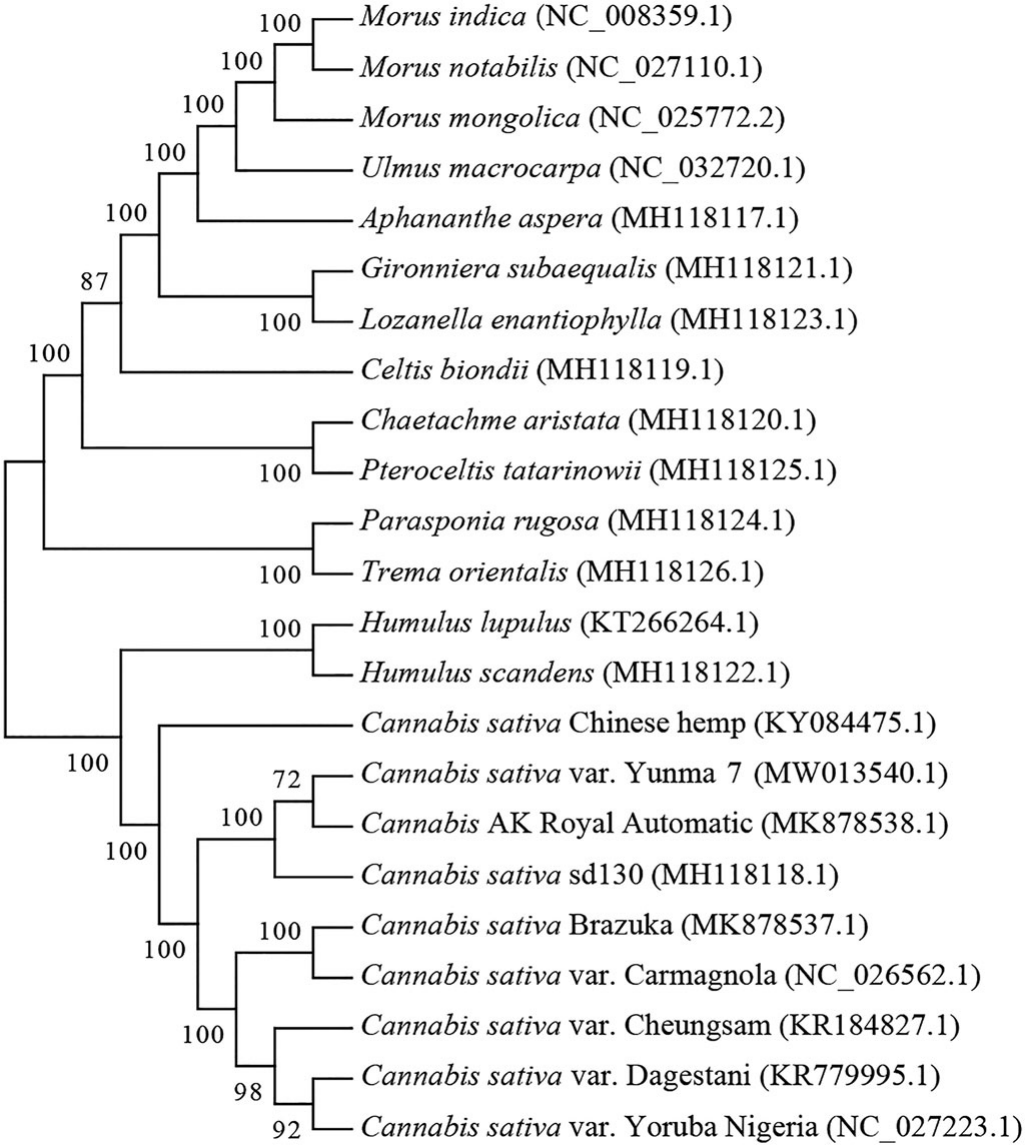
Phylogenetic tree of 23 chloroplast genomes▪.

## Data Availability

The data that support the findings of this study are fully available in SRA (https://www.ncbi.nlm.nih.gov/sra/?term=PRJNA679189) and GenBank (https://www.ncbi.nlm.nih.gov/nuccore/MW013540).

## References

[CIT0001] Deng G, Du GH, Yang Y, Bao YN, Liu FH. 2019. Planting density and fertilization evidently influence the fiber yield of Hemp (*Cannabis sativa* L.). Agronomy. 9(7):368.

[CIT0002] Dierckxsens N, Mardulyn P, Smits G. 2017. NOVOPlasty: de novo assembly of organelle genomes from whole genome data. Nucleic Acids Res. 45(4):e18. 28204566 10.1093/nar/gkw955 PMC5389512

[CIT0003] Doyle JJ, Doyle JL. 1987. A Rapid DNA isolation procedure from small quantities of fresh leaf tissues. Phytochem Bull. 19:11–15.

[CIT0004] Katoh K, Standley DM. 2013. MAFFT multiple sequence alignment software version 7: improvements in performance and usability. Mol Biol Evol. 30(4):772–780. 23329690 10.1093/molbev/mst010 PMC3603318

[CIT0005] Kearse M, Moir R, Wilson A, Stones-Havas S, Cheung M, Sturrock S, Buxton S, Cooper A, Markowitz S, Duran C, et al. 2012. Geneious Basic: an integrated and extendable desktop software platform for the organization and analysis of sequence data. Bioinformatics. 28(12):1647–1649. 22543367 10.1093/bioinformatics/bts199 PMC3371832

[CIT0006] Kojoma M, Seki H, Yoshida S, Muranaka T. 2006. DNA polymorphisms in the tetrahydrocannabinolic acid (THCA) synthase gene in ‘drug-type’ and ‘fiber-type’ *Cannabis sativa* L. Forensic Sci Int. 159(2–3):132–140. 16143478 10.1016/j.forsciint.2005.07.005

[CIT0007] Kumar S, Stecher G, Tamura K. 2016. MEGA7: molecular evolutionary genetics analysis version 7.0 for bigger datasets. Mol Biol Evol. 33(7):1870–1874. 27004904 10.1093/molbev/msw054 PMC8210823

[CIT0008] Luo R, Liu B, Xie Y, Li Z, Huang W, Yuan J, He G, Chen Y, Pan Q, Liu Y, et al. 2012. Soapdenovo2: an empirically improved memory-efficient short-read de novo assembler. Gigascience. 1(1):18. 23587118 10.1186/2047-217X-1-18 PMC3626529

[CIT0009] Marks MD, Tian L, Wenger JP, Omburo SN, Soto-Fuentes W, He J, Gang DR, Weiblen GD, Dixon RA. 2009. Identification of candidate genes affecting Delta9-tetrahydrocannabinol biosynthesis in *Cannabis sativa*. J Exp Bot. 60(13):3715–3726. 19581347 10.1093/jxb/erp210 PMC2736886

[CIT0010] Matielo CBD, Lemos RPM, Sarzi DS, Machado LO, Beise DC, Dobbler PCT, Castro RM, Fett MS, Roesch LFW, Camargo FAdO, et al. 2020. Whole plastome sequences of two drug-type cannabis: insights into the use of plastid in forensic analyses. J Forensic Sci. 65(1):259–265. 31411746 10.1111/1556-4029.14155

[CIT0011] Measham F, Newcombe R, Parker H. 1994. The normalization of recreational drug use amongst young people in north-west England. Br J Sociol. 45(2):287–312. 8055218

[CIT0012] Oh H, Seo B, Lee S, Ahn DH, Jo E, Park JK, Min GS. 2016. Two complete chloroplast genome sequences of *Cannabis sativa* varieties. Mitochondrial DNA A DNA Mapp Seq Anal. 27(4):2835–2837. 26104156 10.3109/19401736.2015.1053117

[CIT0013] Vergara D, White KH, Keepers KG, Kane NC. 2016. The complete chloroplast genomes of *Cannabis sativa* and *Humulus lupulus*. Mitochondrial DNA A DNA Mapp Seq Anal. 27(5):3793–3794. 26329384 10.3109/19401736.2015.1079905

[CIT0014] Wyman SK, Jansen RK, Boore JL. 2004. Automatic annotation of organellar genomes with dogma. Bioinformatics. 20(17):3252–3255. 15180927 10.1093/bioinformatics/bth352

[CIT0015] Zhang H, Jin J, Moore MJ, Yi T, Li D. 2018. Plastome characteristics of Cannabaceae. Plant Divers. 40(3):127–137. 30175293 10.1016/j.pld.2018.04.003 PMC6114266

